# International Academy of Cytology Yokohama System for Reporting Breast Cytology and the ACR Breast Imaging Reporting and Data System (BIRADS): Are they Concordant?

**DOI:** 10.30699/ijp.2024.2028955.3300

**Published:** 2024-10-02

**Authors:** Alka Yadav, Aparna Singh, Sonali Madaan, Mukta Pujani, Sujata Raychaudhuri, Charu Agarwal, Varsha Chauhan, Dipti Sidam, Jyoti Rajpoot, Garima Dhull, Cherry Bansal

**Affiliations:** 1 *Department of Pathology, The Uttar Pradesh University of Medical Sciences, Saifai, Etawah, UP, India*; 2 *Department of Pathology, ESIC Medical College & Hospital, Faridabad, India*; 3 *Department of Radiodiagnosis, ESIC Medical College & Hospital, Faridabad, India*; 4 *Department of Pathology, ESIC PGIMSR, Basaidarapur, New Delhi, India *; 5 *Department of Pathology, Dr. SS Tantia Medical College, Sriganganagar, India*

**Keywords:** IAC Yokohama, breast cytology, BIRADS, ROM, FNAC

## Abstract

**Background & Objective:**

Breast cancer is the leading cause of cancer deaths among women worldwide. Fine needle aspiration cytology (FNAC) and breast sonography have played a pivotal role in the characterization of a breast lump. The main objective of this study was to analyze the correlation between the International Academy of Cytology (IAC) Yokohama for Reporting Breast Fine Needle Aspiration Biopsies (FNAB) and breast imaging reporting and data system (BIRADS) for sonography along with histopathological correlation.

**Methods:**

A total of 135 FNAC specimens were categorized according to the IAC Yokohama system and BIRADS reporting system and their correlation with histopathology wherever possible to calculate the risk of malignancy (ROM).

**Results:**

According to IAC Yokohama categorization, the cases in categories I, II, III, IV, and V were 1,78,8,6 and 42, respectively. Akin to cytology, most of the cases were assigned BIRADS score two followed by score 6, with the Pearson’s correlation coefficient between the IAC Yokohama system for reporting breast cytology and BIRADS scoring system of 1.957 with a P-value < 0.001 (strong correlation). The sensitivity, specificity, PPV, NPV, and DA of FNAC with category III assumed as malignant were 98.9%, 85%, 76.1%, 99.3%, and 89.5%, respectively. Histopathological correlation was available for 90 cases. The ROM for categories II, III, IV, and V was 5.6%,37.5%,100%, and 100%, respectively.

**Conclusion:**

IAC Yokohama system of reporting breast cytopathology and BIRADS serves as a common language of communication between pathologists and clinicians and aid in better stratification of the lesions. Both FNAC (minimally invasive) and ultrasound (non-invasive imaging technique) are diagnostic tools that complement each other for patient diagnosis and management.

## Introduction

Breast cancer is the most common cancer and one of the leading causes of death among women worldwide (1). According to WHO, about 1 lakh new patients with breast cancer are diagnosed annually in India, and an estimated 70,218 Indian women die due to breast cancer every year (2). Most breast cancers present as palpable lumps, inflammatory lesions, nipple secretions, or mammographic abnormalities. Preoperative pathology diagnosis constitutes an essential part of the workup of breast lesions wherein radiology and cytology play a crucial role (3). Whenever there is a discrepancy between clinical examination, breast fine needle aspiration cytology (FNAC), and sonography, a biopsy is recommended to make a final diagnosis. 

BIRADS stands for “Breast Imaging Reporting and Data System. The American College of Radiology proposed to provide a widely accepted lexicon and reporting scheme for imaging of the breast. It includes seven categories with recommendations for each category and the likelihood of cancer, namely 0. Incomplete assessment, 1. normal, 2. benign finding, 3. probably benign, 4. suspicious abnormality, 5- highly suspicious of malignancy, and 6- known, biopsy-proven malignancy (4). 

A triple assessment approach is advocated in many countries for assessing a breast mass, combining clinical, radiological, and cytopathological information, thereby ensuring an accurate diagnosis and patient management (5). 

Fine needle aspiration cytology (FNAC) is a simple, rapid, cost-effective, minimally invasive, accurate procedure that plays a pivotal role in the early diagnosis and categorization of a breast lump into benign or malignant. The role of FNAC in diagnosing breast lesions is crucial however histopathology remains the gold standard. Cyto-histopathological correlation is of great relevance and increases precision (6). 

 Differentiation is not possible in all cases due to significant cytomorphologic overlap between benign and malignant breast lesions (7). To address these grey zones and to bring a degree of uniformity to the reporting system, in 1996, the National Cancer Institute (NCI) proposed five diagnostic categories (8). Ever since, the use of FNAC in the evaluation of breast lesions has changed substantially over the period of 20 years, mainly due to changes in screening programs and available treatments and the recent preference for core needle biopsy (CNB). Recently, the cytological diagnosis has been categorized under the widely accepted five-tier reporting format for breast lesions laid down by The International Academy of Cytology (IAC, Yokohama 2016), i.e. Category I (insufficient material), Category II (benign), Category III (atypical, probably benign), Category IV (suspicious, probably in situ or invasive carcinoma) and Category V (malignant) (7). 

An extensive literature search revealed various studies on the spectrum of breast lesions as per IAC standardized categories (9–11). However, there is a dearth of literature on the correlation between IAC Yokohama subcategories on cytology and BIRADS categories (12). The present study was conducted to categorize the breast lesions according to the BIRADS system on sonography, IAC Yokohama system on cytology, and cyto-histological correlation wherever feasible and to evaluate the risk of malignancy for each category of the IAC Yokohama system as well as to calculate the sensitivity, specificity, positive predictive value (PPV), negative predictive value (NPV) and diagnostic accuracy for all categories.

## Materials and Methods

The present study was a prospective cross-sectional study conducted at departments of Pathology and Radiology, ESIC Medical College, and Hospital Faridabad over a period of six months from January to June 2023. The study was conducted following approval by the institutional ethical committee.

All Patients presenting with breast lumps who underwent both ultrasonography and Fine needle aspiration cytology for the evaluation of the lesion were included in the study. The patients with breast lumps who had normal sonographic findings (BIRADS-1) or with a history of any previous breast surgery/recurrent lump were excluded.

The present study is a tertiary care hospital-based study on patients referred to the Department of Pathology for routine FNAC of breast lumps. Clinical details like age, size, site, duration of lump, and symptoms were recorded for each case. For FNAC, patients were made to lie down. The lump was palpated, the area was sterilized, and FNAC was performed using a 22/23-gauge needle. In all cases, the FNAC was performed using the palpation-guided method on the first attempt. The repeat FNAC was performed under sonographic guidance if the material was not obtained. Air-dried smears were stained by Giemsa and alcohol-fixed smears by Papanicolaou. After a thorough cytological examination, the lesions were categorized as per IAC, Yokohama 2016.

A radiologist performed an ultrasonography of the breast masses in the department of radiodiagnosis. The following features were evaluated on the scans- Shape of the lesion - Round/Oval or irregular; Margins of the lesion- well circumscribed/non-circumscribed; Dimensions/ Width-AP ratio>1.4 or =1.4 and Echogenicity – Hyper /Iso or Hypoechoic. Diagnosis was established considering these four features, and a BIRADS score was assigned.

Histopathological correlation was also done wherever possible. The risk of malignancy (ROM) was calculated for each category using the formula, number of confirmed malignant cases to the total number of cases in every diagnostic category.

Statistical analysis was executed using Microsoft Excel 2011 and SPSS software. Sensitivity, specificity, positive predictive value (PPV), negative predictive value (NPV), and diagnostic accuracy were calculated for all categories. Pearson’s correlation coefficient was calculated to study the concordance between the IAC Yokohama system for reporting breast cytology and the BIRADS scoring system.

## Results

A total of 135 cases were included in the study, which was categorized according to the IAC Yokohama system on breast cytology and the BIRADS score assessment on sonography. The mean age of the patients was 33 years, while the mean size of the breast lump was 4.5 cm. All but one patient were females. The clinicopathological data of the study population is depicted in Table 1.

**Fig. 1:**
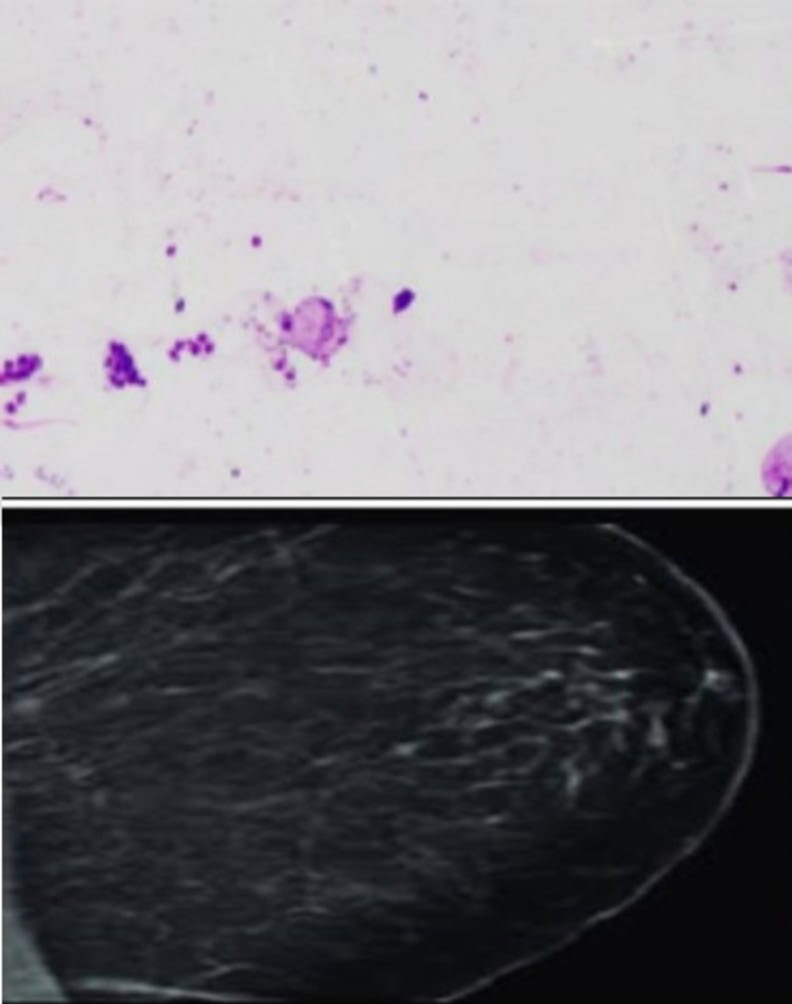
IAC Category 1 showing scant cellularity, very few ductal epithelial cells; BIRADS 1 showing enlargement of fibroglandular and fibrofatty tissue only.

**Fig. 2:**
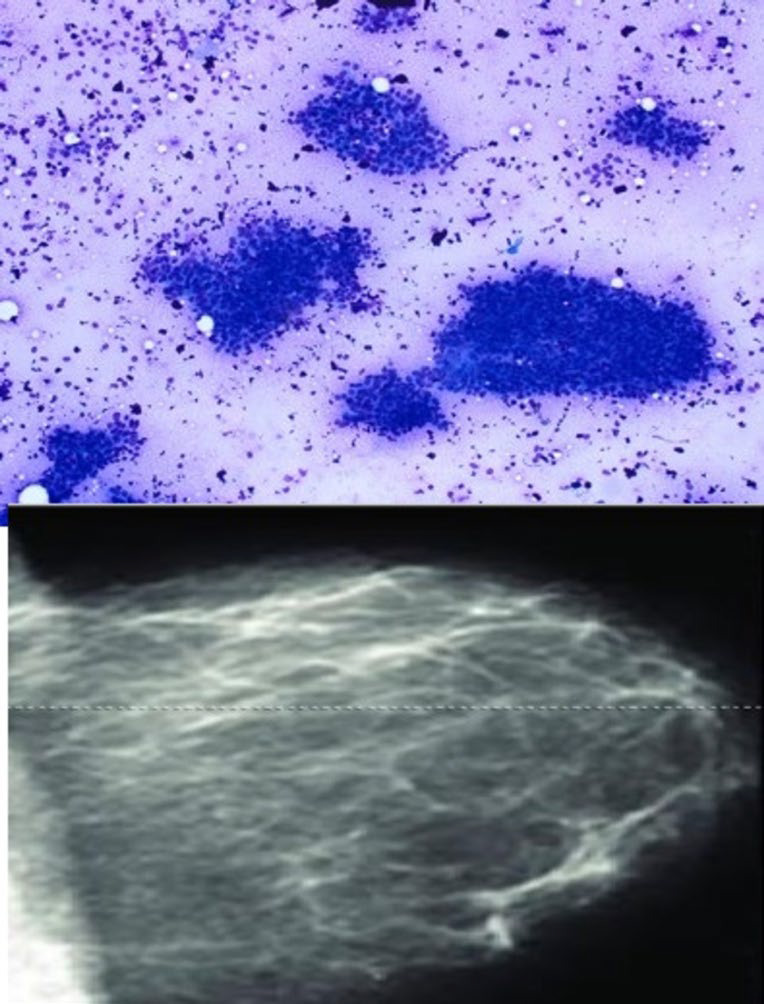
IAC category 2 showing tightly cohesive clusters of ductal epithelial cells along with the myoepithelial cells. Background showing bare bipolar nuclei; BIRADS 2 -Image showing few scattered heterogeneous density areas.

**Fig. 3:**
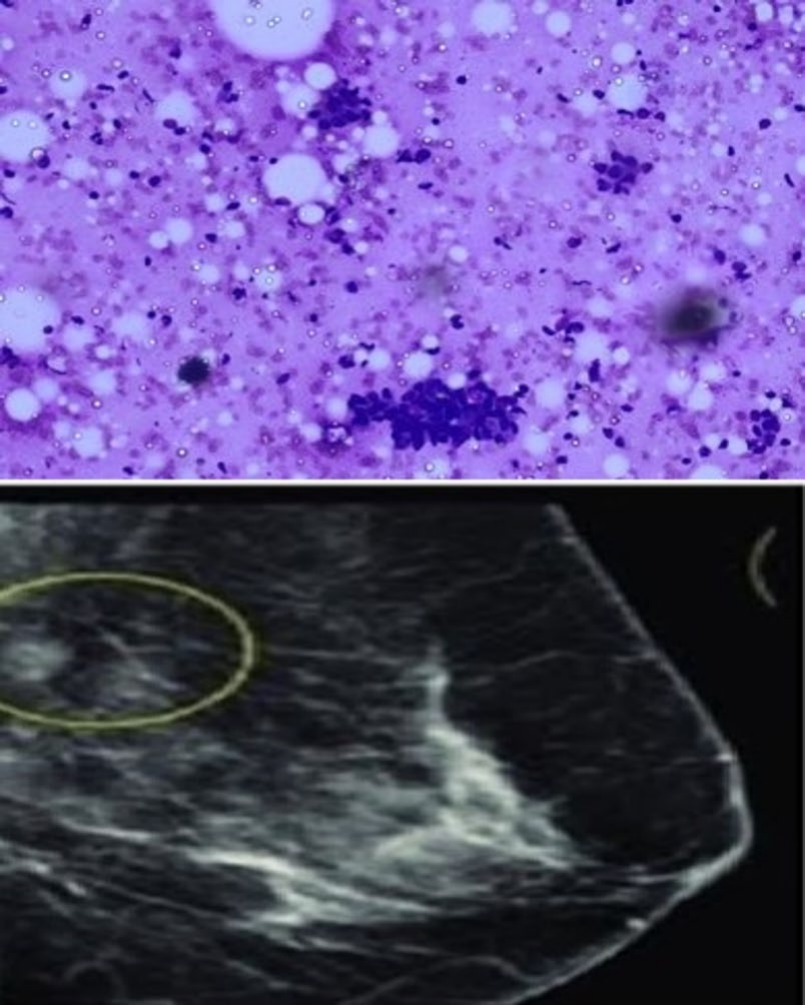
IAC category 3 -Smear showing few dyscohesive clusters of ductal epithelial cells and few bare bipolar nuclei; Image of BIRADS 3 showing focal area of heterogeneous density.

**Fig. 4:**
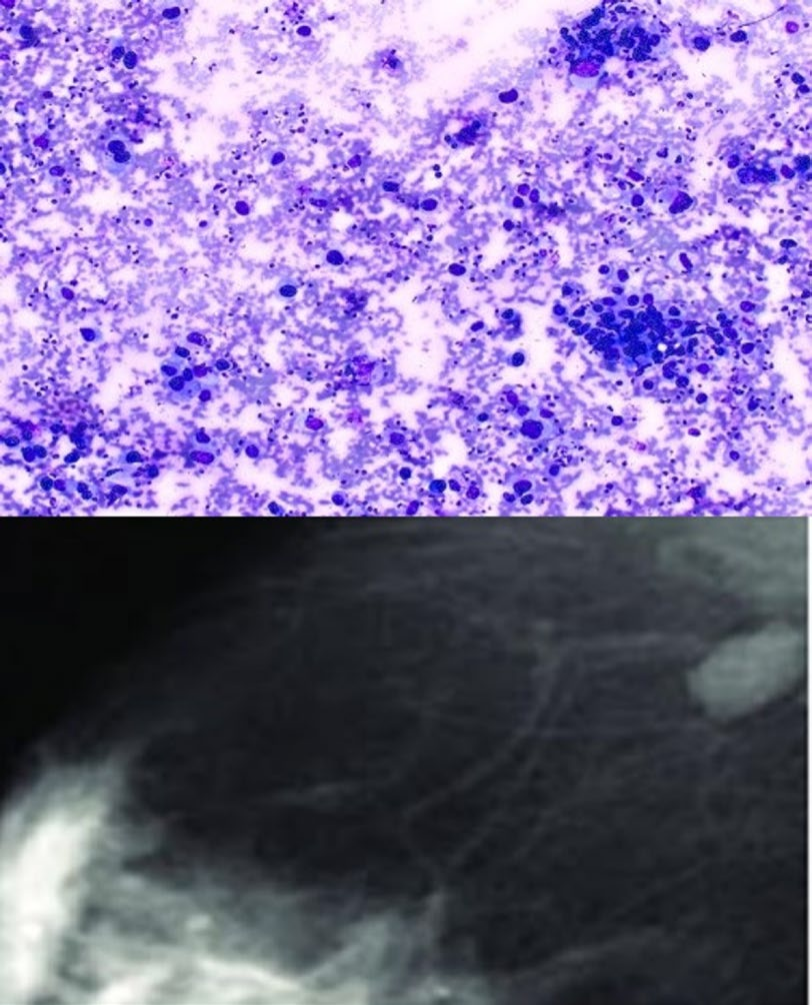
IAC category 4-showing loosely cohesive clusters and singly scattered showing anisonucleosis; Image of BIRADS 4 shows heterogeneous enhancing areas.

**Fig. 5:**
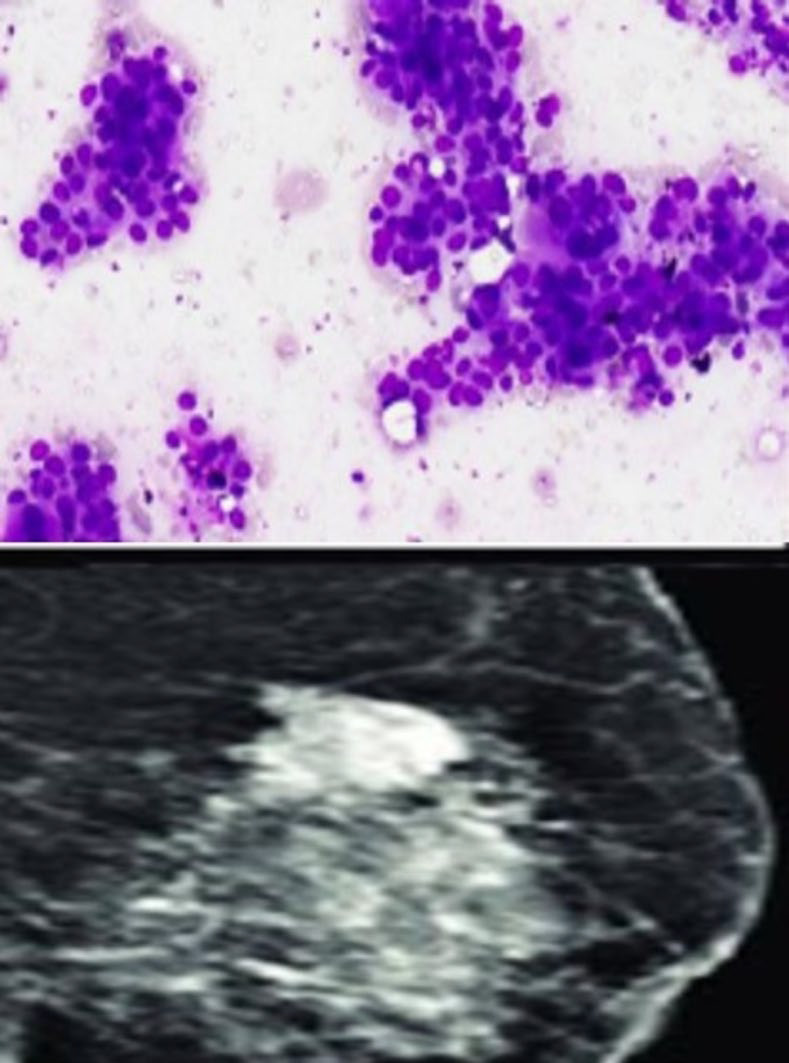
IAC category 5 showing loosely cohesive clusters showing anisonucleosis; BIRADS 6 image showing heterogenous areas with microcalcifications.

The correlation between the Breast Imaging-Reporting and Data System (BIRADS) and the Yokohama International Academy of cytology grading for breast lesions is shown in Table 2. The number of cases in Category I (insufficient material), Category II (benign), Category III (atypical, probably benign), Category IV (suspicious, probably in situ or invasive carcinoma) and Category V (malignant) were 1,78,8,6 and 42 respectively. Regarding cytology, most cases were assigned BIRADS score 2 followed by BIRADS score 6 (Figures 1, 2, 3, 4, and 5).

**Table 1:** Clinico-pathological parameters of all of the patients in the study group (n=135).

Clinicopathological Parameter	Category	No.	Percentage (%)
Age(years)	<20	39	28.89
	21-40	54	40
	41-60	34	25.18
	>60	8	5.93
Tumor Size	≤2 cm	29	21.5
	2-5cm	86	63.7
	> 5 cm	20	14.81
Gender	Male	1	0.07
	Female	134	99.26
Site of Lesion (Laterality)	Left	80	59.26
	Right	55	40.74
Radiological Categories (USG)	BIRADS 1	0	0
	BIRADS 2	78	57.78
	BIRADS 3	4	2.96
	BIRADS 4	5	3.7
	BIRADS 5	16	11.85
	BIRADS 6	32	23.7
Cytological Categories (IAC Yokohama classification 2016)	Category 1 Insufficient	1	0.07
	Category 2 Benign	78	57.78
	Category 3 Atypical	8	5.93
	Category 4 Suspicious of Malignancy	6	4.44
	Category 5 Malignant	42	31.11
Histopathological Diagnosis (n=90)	Fibrocystic disease	2	1.48
	Fibroadenoma	39	28.89
	IDC	49	36.29

**Table 2:** Correlation of Breast Imaging-Reporting and Data System (BIRADS) and Yokohama International Academy of Cytology grading for breast lesions.

IAC BIRADS	Category 1 Inadequate	Category 2 Benign	Category 3 Atypical	Category 4 Suspicious of Malignancy	Category 5 Malignant	Total
BIRADS 1	-	2	-	-	-	2
BIRADS 2	1	76	5	-	-	82
BIRADS 3	-		3	3	-	6
BIRADS 4	-	-	-	3		3
BIRADS 5	-	-	-	-	4	4
BIRADS 6	-	-	-	-	38	38
Total	**1**	**78**	**8**	**6**	**42**	**135**

**Table 3:** Correlation of Yokohama International Academy of Cytology grading with the histopathologic features of breast lesions

	Histopathology available	Histopathological Diagnosis		Risk of Malignancy
		Benign	Malignant	
Category 1 (1) Inadequate	0	0	0	-
Category 2 (78) Benign	38	36	2	5.26%
Category 3 (8) Atypical	8	5	3	37.5%
Category 4 (6) Suspicious	6	0	6	100%
Category 5 (42) Malignant	38	0	38	100%
Total	**90**	**41**	**49**	

**Fig. 6:**
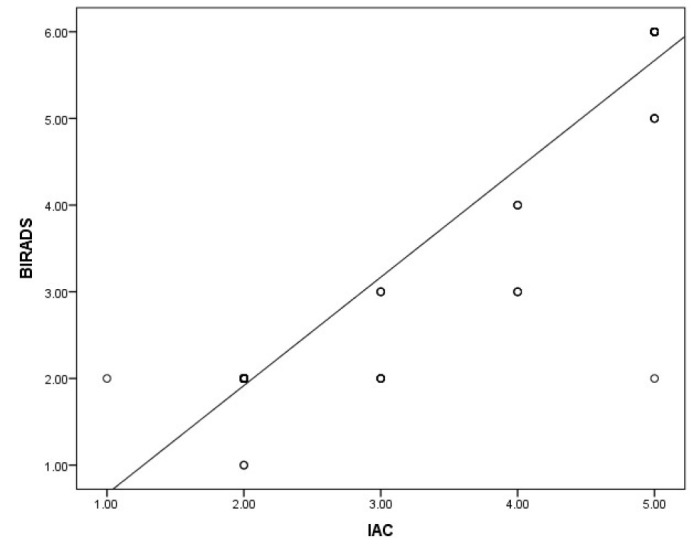
The chart showing correlation between IAC Yokohama system and BIRADS Scoring

Pearson’s coefficient to study the correlation between the IAC Yokohama system for reporting breast cytology and the BIRADS scoring system was found to be 1.957 with a P-value< 0.001, indicating a very strong correlation (Figure 6). 

Histopathological correlation was done in 90 cases where biopsy/ surgical resection was performed. Table 3 shows the correlation between cytological and histopathological diagnosis. In the present study, the sensitivity, specificity, positive predictive value (PPV), negative predictive value (NPV), and accuracy of fine needle aspiration biopsy (FNAB) with category III assumed as malignant was 98.9%, 85%, 76.1%, 99.3%, and 89.5%, respectively.

**Table 4:** Comparative Analysis of risk of malignancy (ROM) of each IAC Yokohama Category for the breast cytology

Study	Year	Category 1	Category 2	Category 3	Category 4	Category 5
Wong et al.^25^	2019	2.6	1.7	15.7	84.6	99.5
Hoda et al.^26^ (Meta-analysis)	2019	30.3	4.7	51.5	85.4	98.7
Kamatar *et al*.^20^	2019	0	4	66	83	99
Chauhan et al.^15^	2019	0	0	25	80	100
Montezuma D *et al*.^27^	2019	4.8	1.4	13	97.1	100
Apuroopa et al.^28^	2020	5	1.2	12.5	93.65	100
Deshpande et al^21^	2021	0	2.04	10.81	85.71	100
Ahuja et al.^29^	2021	5	1.5	17.4	81.8	100
Nargund et al.^30^	2021	7.6	15.26	65.38	83.33	99.18
Agrawal et al.^22^	2021	-	5	25	71	99.7
Marabi et al^35^	2021	8.8	0.5	22.6	89.2	100
Sundar et al.^31^	2022	38	0.6	21.9	100	97
Aithmia et al.^23^	2022	0	2.27	50	50	100
Surekha et al.^24^	2022	0	1.1	6.2	81	98
Niaz et al.^32^	2022	45.45	10.3	30.6	82.79	99.34
Nikas et al.^33^ (Meta-analysis)	2023	17	1	20	86	100
Shankar et al^36^	2023	10	2.5	44.4	100	100
Dogra et al^37^	2023	100	0	55.5	100	100
Yadav et al^38^	2024	16.6	1.02	19.04	75	100
Present Study	2024	-	5.2	37.5	100	100

## Discussion

Fine needle aspiration cytology is an accurate diagnostic test for breast lesions, offering the added advantage of low costing and rapid reporting (13). The high accuracy of breast FNAB in diagnosis of malignant disease confirms its suitability as a first-line diagnostic test. IAC Yokohama reporting system is a standardized, universally practiced approach providing a definitive diagnostic category for breast cytology reporting and furnishes useful information to the surgeon including ROM.

In our study population, we categorized breast lump according to Yokohama reporting system and compared the results with other studies. Most frequent age group in our study was in the range of 21-45 years, similar to present study conducted by Verma *et.al* (14) Most of the patients had left breast lump similar to Chauhan *et al *(15). On cytology, most of the patients in our study were placed in category II, benign, followed by category V, malignant, category III, and category IV; few cases were in category I. These findings were in accordance with Muddegowda *et al. *(16), Mehra* et al. *(17), Daramola* et al. *(18), and Panjvani* et al.* (19).

Cyto-histopathological correlation was available in 90 cases for which the risk of malignancy was calculated. In the present study, the ROM for categories II, III, IV, and V was 5.6%,37.5%,100%, and 100%, respectively, which is comparable to many studies, including Kamatar *et al*. (20), Chauhan *et al.* (15) Deshpande* et al.* (21), Agrawal *et al.* (22), Aithmia *et al.* (23), Surekha *et al.* (24). A comparative analysis of ROM of IAC Yokohama categories across various studies from worldwide literature is depicted in Table 4. 

Nikas *et al. (*33*) conducted the first meta-analysis of the *pooled risk of malignancy for the various IAC Yokohama system categories. The categories I (Insufficient), II (Benign), II (Atypical), IV (Suspicious), and V (Malignant) had a pooled ROM of 17% (95% CI, 10%-28%), 1% (95% CI, 1%-3%), 20% (95% CI, 17%-23%), 86% (95% CI, 79%-92%), and 100% (95% CI, 99%-100%), respectively. The sensitivity was 91% (95% CI, 87.6%-93.5%), and the false-positive rate was 2.33% (95% CI, 1.30-4.14%) when both "Suspicious" and "Malignant" categories were collectively considered as cytologically positive. A summary receiver operating characteristic curve was constructed and the pooled area under the curve was 97.3%, while the pooled diagnostic odds ratio was 564 (95% CI, 264-1,206), implying a high level of diagnostic accuracy.

There was no histopathology available for category I case, so no correlation was done in this category. Few studies showed higher ROM for category 1, however overall risk of malignancy was low on comparing with other categories. This could be due to non-representative sampling on aspiration cytology; therefore, the importance of multiple passes and careful examination needs to be re-emphasized for the category 1 lesions.

The 2 cases in category II which came out to be malignant on histopathology as the lesion was small in size and was freely mobile, however the cellularity was high and it was considered as cellular fibroadenoma. The 5 cases in atypical category led to misdiagnosis because of their cellularity; these lesions are difficult to differentiate on cytomorphology. The presence of loose cohesive clusters with mild atypia and very few benign bipolar nuclei scant stromal fragments in the background led to the categorization benign proliferative breast disease with atypia. On histopathology they were diagnosed as cellular fibroadenoma.

Ultrasound is a very useful imaging tool in predicting the likelihood of cancer among breast lesions. BIRADS proposed by American College of Radiology is a universally accepted, standardized reporting system for imaging of the breast comprising of seven categories with the probability of cancer and clinical recommendations for each category. In addition, recent developments in both cytology and imaging like Immunocytochemistry, imaging guided FNAC and Doppler in sonomammography may further improve their accuracy (28).

In the present study the sensitivity, specificity, positive predictive value (PPV), negative predictive value (NPV), and accuracy of FNAB with category III assumed as malignant were 98.9%, 85%, 76.1%, 99.3%, and 89.5%, respectively. The results are comparable with Montezuma D *et al. *(27), and Moschetta M* et al.* (34). With category III assumed as benign, these indices were 90.8%, 98.9%, 97.5%, 95.7%, and 96.2%, respectively. The sensitivity, specificity, PPV, NPV, and accuracy of BI-RADS were 91.5%, 81.9%, 72%, 95%, and 85.1%, respectively.

The limitations of the current study were the small sample size and the fact that histopathological follow-up was available in fewer cases. More multicentre studies with large sample sizes need to be conducted to evaluate the validity of the Yokohama system for reporting breast cytology.

## Conclusion 

The present study reaffirms the complementary role of FNAC (IAC Yokohama reporting system) in conjunction with imaging (BIRADS standardized scheme), demonstrating a significant correlation between the two methods and with histopathology. This strong correlation provides a solid foundation for clinical decision-making in breast lesions. It also enhances the overall reporting process, fosters better communication between cytopathologists, radiologists, and surgeons, and ultimately leads to improved patient care. 
